# Local application of silver nitrate as an adjuvant treatment before deep lamellar keratoplasty for fungal keratitis poorly responsive to medical treatment

**DOI:** 10.3389/fmed.2023.1292701

**Published:** 2024-01-22

**Authors:** Gang Ding, Xin Gao, Yue Tan, Zhongkai Hao, Ximing Wang, Chenming Zhang, Aijun Deng

**Affiliations:** ^1^Department of Ophthalmology, The Jinan Second People's Hospital, Jinan, China; ^2^Department of Ophthalmology, School of Clinical Medicine, Weifang Medical University, Weifang, China; ^3^Department of Ophthalmology, Affiliated Hospital of Weifang Medical University, Weifang, China

**Keywords:** fungal keratitis, deep lamellar keratoplasty, silver nitrate, adjuvant treatment, efficacy

## Abstract

**Objective:**

The purpose of this study is to evaluate the efficacy and safety of the local application of silver nitrate (LASN) as an adjuvant treatment before deep lamellar keratoplasty (DLKP) for fungal keratitis responding poorly to medical treatment.

**Methods:**

A total of 12 patients (12 eyes) with fungal keratitis responding poorly to medical treatment (for at least 2 weeks) were included. LASN was performed using 2% silver nitrate, the ulcer was cleaned and debrided, and then, the silver nitrate cotton stick was applied to the surface of the ulcer for a few seconds. The effect of LASN was recorded. The number of hyphae before and after treatment was determined by confocal microscope. After the condition of the ulcer improved, DLKP was performed. Fungal recurrence, best-corrected visual acuity (BCVA), loose sutures, and endothelial cell density (ECD) were recorded in detail.

**Results:**

Clinical resolution of corneal infiltration and edema was observed, and the ulcer boundary became clear in all 12 patients after 7–9 days of LASN. Confocal microscopy showed that the number of hyphae was significantly reduced. Ocular pain peaked on days 1 and 2 after treatment, and 9 patients (75%, day 1) and 1 patient (8.3%, day 2) required oral pain medication. During the follow-up period after DLKP, no fungal recurrence and loose sutures were observed. After the operation, the BCVA of all patients improved. The mean corneal ECD was 2,166.83 ± 119.75 cells/mm^2^.

**Conclusion:**

The LASN was safe and effective and can be well tolerated by patients. Eye pain can be relieved quickly. LASN as an adjuvant treatment before DLKP might be a promising therapeutic strategy.

## 1 Introduction

Fungal keratitis is a potentially blinding infection of the cornea that afflicts diverse patient populations worldwide (1). Fungal keratitis is painful and may require hospitalization for intensive medical treatment and/or surgical interventions. Treatment of fungal keratitis with systemic and topical antifungal agents has been only partially successful due to the high resistance to antifungal agents (2). Natamycin and voriconazole are not sufficient for treating non-responsive fungal keratitis due to their limited efficacy (3). DLKP is effective in treating fungal keratitis in patients who are non-responsive to medical therapy (4). However, the recurrence of fungal keratitis remains to be the main reason for graft failure.

Silver has been increasingly used as a component of various medical devices with the intention of reducing infections. Silver nitrate was used topically throughout the 1800s for the treatment of burns, ulcerations, and wound infections (5). The beneficial effects of silver nitrate in reducing or preventing infection have been observed in the topical treatment of ophthalmia neonatorum (6). In addition, the silver nitrate solution also has antimicrobial properties and can reduce the microbial load (7). Silver compounds were widely used as effective antimicrobial agents to combat pathogens (bacteria, viruses, and eukaryotic microorganisms) in the clinic. Silver cations (Ag+) were used to treat burns, wounds, and ulcers (8). Ag+ was usually found in compounds, e.g., silver nitrate and silver sulfadiazine.

Nevertheless, regular preoperative treatment has not been routinely recommended before DLKP. Silver nitrate has a good antibacterial effect, but it is rarely used in fungal keratitis. In our institution, silver nitrate has been used for many years to treat keratitis with satisfactory efficacy (9). In this study, we employed LASN as an adjuvant treatment before DLKP for fungal keratitis and a favorable clinical outcome was achieved.

## 2 Materials and methods

### 2.1 patients

This retrospective study included data from 12 patients (12 eyes) with fungal keratitis who were enrolled at the Jinan Second People's Hospital (Jinan Eye Hospital) between March 2021 and January 2022. The diagnosis of fungal infection was made on the basis of clinical evaluation, positive smear, cultures of the fungus, and confocal microscopy results. The study protocol was approved by the Institutional Review Board of the Ethics Committee of Jinan Second People's Hospital (Jinan Eye Hospital) (No. JNEYE20210205). Each patient gave informed written consent for participation and local application of silver nitrate and surgical management.

The inclusion criteria: 1. the ulcer showing no change/increase in the size of the epithelial defect and aggravation of stromal infiltrate or increase in hypopyon on standard topical natamycin 5% (once/hour) and voriconazole 1% eye drops (once/hour) therapy for at least 2 weeks; 2. thickness of the infiltrations being larger than one-fifth but smaller than four-fifth of the corneal thickness, as observed by cornea optical coherence tomography (OCT); 3. the ulcer affecting the optical zone; 4. the infiltrate diameter being over 6 mm. The exclusion criterias were as follows: 1. corneal perforation; 2. extensive corneal involvement till Descemet's membrane.

### 2.2 Local application of silver nitrate

**Figure 1:**
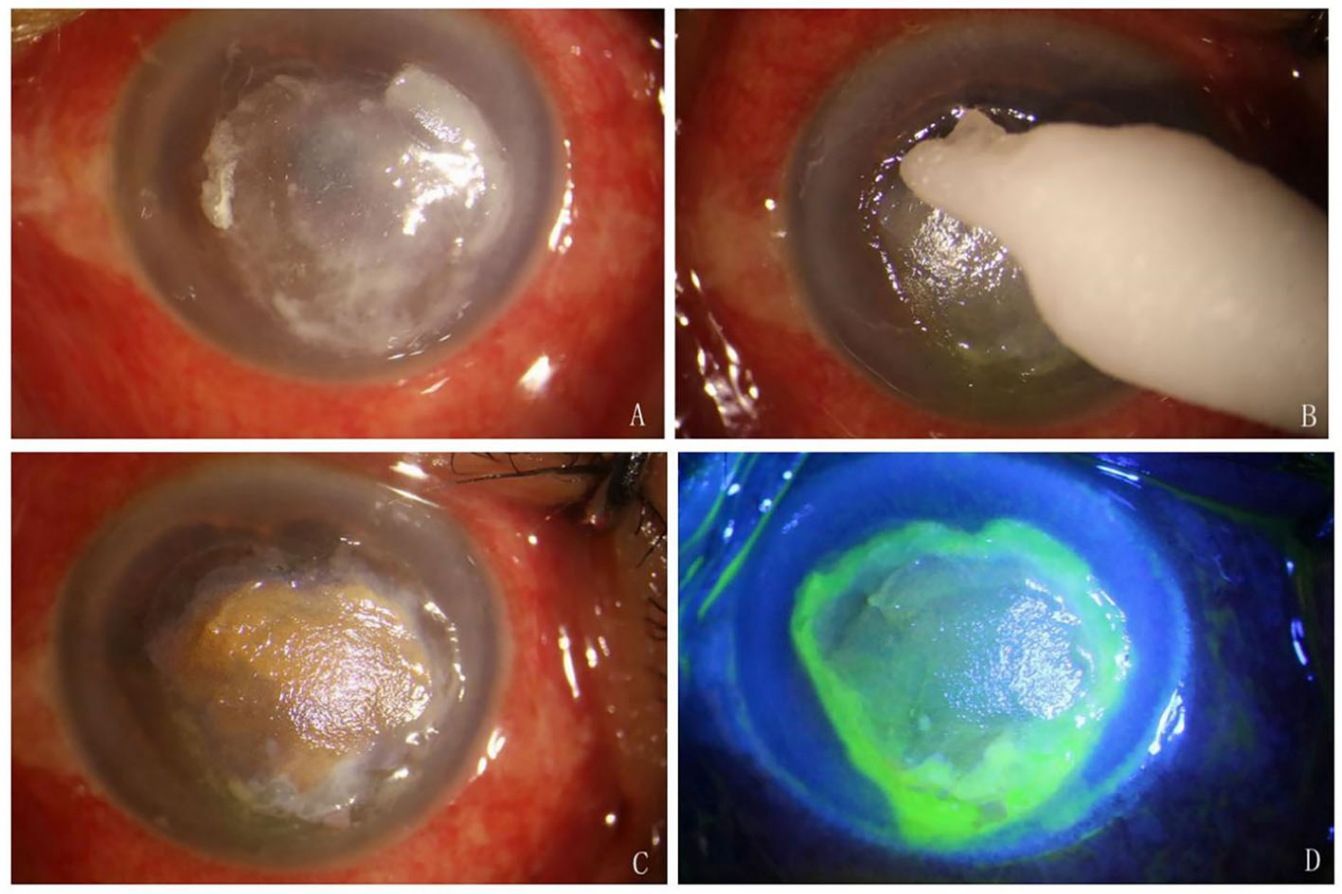
Procedures of LASN. **(A)** The ulcer was cleaned and debrided. **(B)** Silver nitrate cotton stick was applied to the surface of the ulcer. **(C)** A brown precipitate was formed. **(D)** Fluorescein sodium staining showed that the cornea around the ulcer and conjunctiva was negative.

LASN was performed using 2% silver nitrate. We used silver nitrate in the outpatient operating room. Proparacaine hydrochloride eye drops were used for surface anesthesia before the procedure. The ulcer was cleaned and debrided. Cotton sticks were soaked with silver nitrate solution and were applied to the surface of the ulcer for a few seconds until a white or brown precipitate was formed (Figure 1). The duration of the LASN was 15 s. It was spread to the entire ulcer. The procedure was completed under a slit lamp microscope. Precautions were taken in all applications for the cotton sticks not to contact the surrounding healthy cornea. We used a lid opener during the treatment, which facilitates operation and avoids contact with the eyelids and conjunctiva. Conjunctival sac irrigation was performed using sterile water immediately after local application. The procedure of LASN was performed once.

A slit lamp biomicroscope was used to assess the outcomes of LASN. The number of hyphae before and after treatment was assessed by confocal microscope. The presence of chemical conjunctivitis and pain was also recorded. Self-reported pain was recorded using a visual analog scale (VAS range, 0–10). Effective treatment was defined as partially healed ulcers, alleviated inflammatory responses, relieved lesions, and no fungal hyphae or reduction in hyphae, and reduction of empyema in the anterior chamber. Ineffective treatment was defined by increased numbers of fungal hyphae, aggravated area and depth of the ulcer (10), and increase in empyema in the anterior chamber.

### 2.3 Surgical procedures of DLKP

All operations were completed by one surgeon. After general anesthesia, trephination was partially removed with a Hessburg-Barron trephine whose diameter was larger than 0.25 mm of the infiltration border, and then, the anterior diseased stroma was excised. Deeper layer-by-layer stromal dissection was carried out using a surgical knife until it was close to the Descemet's membrane and showed that the residual corneal stroma was transparent. The operation was assisted by intraoperative optical coherence tomography (iOCT). The host bed was prepared. The donor cornea was trephined by a size of 0.25 mm larger than the host bed. Finally, the corneal graft was sutured to the recipient using 16 interrupted 10/0 nylon sutures (Mani, Tochigi, Japan). At the end of the operation, erythromycin eye ointment was used to prevent infection.

### 2.4 Postoperative treatment

After the operation, patients were prescribed topical 1% voriconazole eye drops or (combined with) 5% natamycin eye drops and 0.1% tacrolimus four times/day, and these regimens were gradually reduced depending on the condition of the illness. Corneal sutures were removed according to the patients' condition.

All cases were followed up for at least 6 months. During the follow-up, patients' condition, fungal recurrence, BCVA, loose sutures, and ECD were recorded in detail.

## 3 Result

### 3.1 Baseline demographic and clinical characteristics of the patients

Among the 12 patients, there were 4 males and 8 females. The mean age of the patients was 56.42±6.89 years old. The infiltrate diameter was 7.35 ± 0.65 mm. The depth of infiltrate was 1/4–3/4. Of all cases, only five of them had hypopyon. The KOH smear and corneal scrapings were positive in all patients. *Fusarium* was isolated in 10 patients, *Aspergillus* was isolated in 1 patient, and *Alternaria* was isolated in 1 patient (Table 1).

**Table 1:** Baseline demographic and clinical characteristics of the patients.

**Case**	**Gender**	**Age**	**Risk factor for fungal keratitis**	^*^Diameter	**Depth**	**Hypopyon**	**Smear**	**Confocal**	**Pathogen**
				**(mm)**					
1	Female	59	Vegetative trauma	7.25	2/5	Yes	Positive	Positive	*Fusarium*
2	Male	51	Vegetative trauma	7.75	3/4	No	Positive	Positive	*Fusarium*
3	Female	67	Vegetative trauma	7.5	1/2	No	Positive	Positive	*Fusarium*
4	Female	52	Vegetative trauma	8.75	3/5	Yes	Positive	Positive	*Fusarium*
5	Male	50	Probably trauma	6.75	1/3	No	Positive	Positive	*Fusarium*
			(No clear history)						
6	Male	46	Vegetative trauma	6.5	2/3	No	Positive	Positive	*Aspergillus*
7	Male	49	Probably trauma	7	1/4	No	Positive	Positive	*Fusarium*
			(No clear history)						
8	Female	58	Probably trauma	7.5	3/5	Yes	Positive	Positive	*Fusarium*
			(No clear history)						
9	Female	63	Vegetative trauma	6.75	2/5	No	Positive	Positive	*Fusarium*
10	Female	56	Vegetative trauma	8.25	3/4	Yes	Positive	Positive	*Fusarium*
11	Female	60	Probably trauma	7.25	2/5	Yes	Positive	Positive	*Alternaria*
			(No clear history)						
12	Female	66	Vegetative trauma	7	1/2	No	Positive	Positive	*Fusarium*

^*^Diameter: ulcer maximum diameter.

### 3.2 Outcomes of LASN

**Figure 2:**
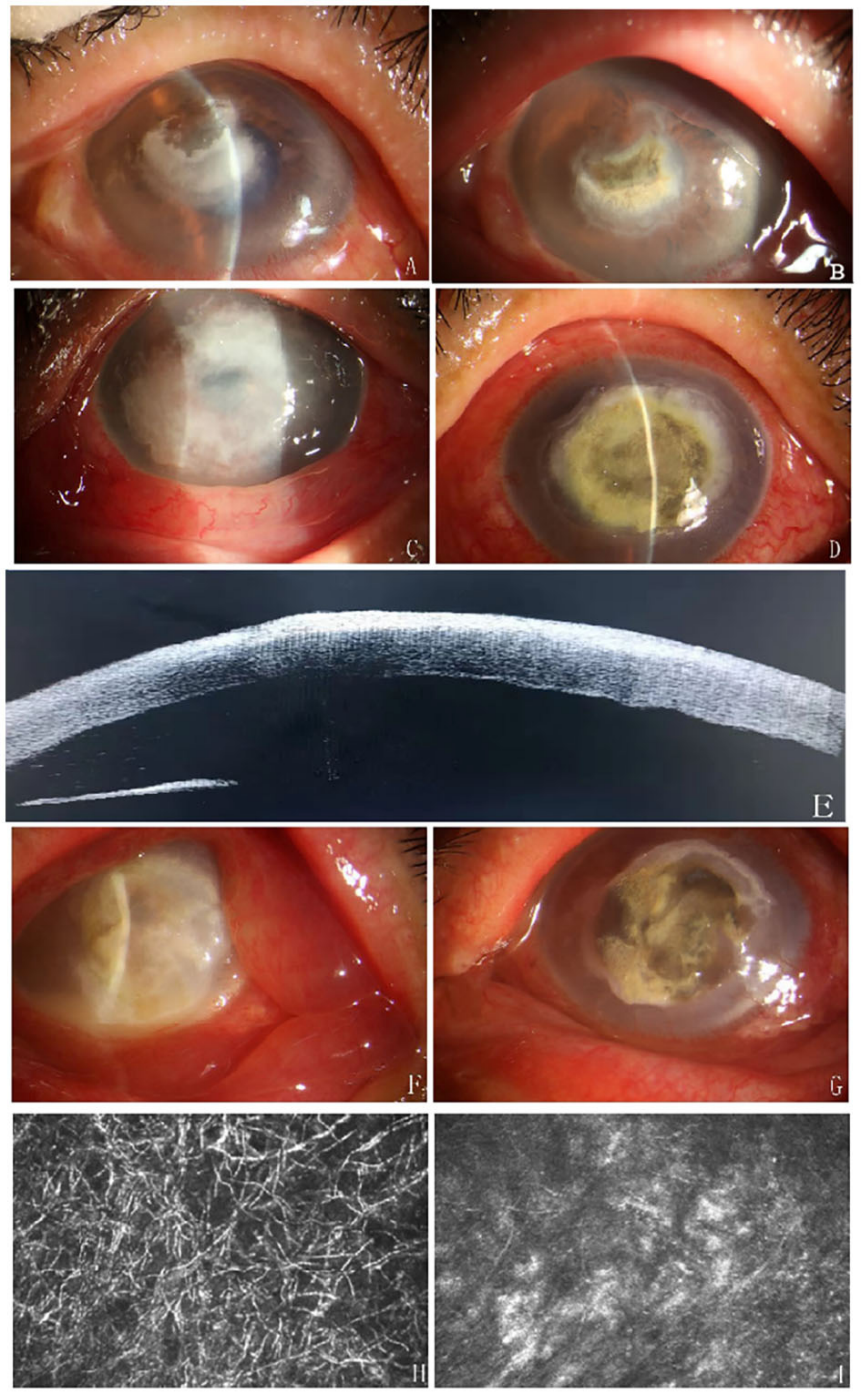
Examples of efficacy after treatment. Case 1, Central corneal infiltration with feathery margins **(A)**, clinical photograph shows an effective response to silver nitrate therapy: the size of the ulcer was reduced and infiltration was alleviated 7 days after treatment **(B)**. Case 2, A large corneal ulcer with feathery margins **(C)** and the patient responded effectively to the LASN on day 8 **(D)**. The patent's OCT as shown and the depth of lesion infiltration reached the shallow to medium stromal layer **(E)**. Case 3 had a serious fungal infection with hypopyon **(F)**, after LASN, the ulcer had a clear boundary, the peripheral corneal edema was reduced, and the empyema disappeared **(G)**. IVCM pictures of the patient (case 3) showed a large number of hyphae **(H)** and the number of hyphae was significantly reduced on day 7 after treatment **(I)**.

Therapeutic efficacy is shown in Figure 2. All patients were effective. Clinical resolution of corneal infiltration and edema was observed after 7–9 days of LASN. After treatment with silver nitrate, the ulcer boundary became clear in 12 patients, and the size of the ulcer was reduced in 4 patients. Hypopyon was observed in five eyes before the treatment and was absorbed after 6.20±0.83 days of LASN. Confocal microscopy showed that the number of hyphae was reduced on days 7–9 after treatment. DLKP surgery was performed 8.08 ± 0.79 days after LASN treatment (Table 2).

**Table 2:** Clinical characteristics and outcomes after DLKP.

**Case**	**Pre-operative**		**Days from LASN to surgery**	**Post-operative**			
	**BCVA (LogMAR)**	**Donor graft size (mm)**		**BCVA (LogMAR)** ^*^	**ECD (cell/mm** ^2^ **)** ^*^	**Recurrence** ^*^	**Loose sutures** ^#^
1	2.2	7.5	8	0.6	2,223	No	No
2	0.9	8	9	0.5	2,154	No	No
3	2.2	7.75	8	0.7	1,992	No	No
4	LP	9	9	0.9	2,072	No	No
5	2.3	7	7	0.4	2,340	No	No
6	2.1	6.75	7	0.3	2,176	No	No
7	2.2	7.25	8	0.6	2,089	No	No
8	LP	7.75	8	0.5	2,302	No	No
9	2.3	7	9	0.4	2,287	No	No
10	2.4	8.5	9	0.9	1,959	No	No
11	2.1	7.5	8	0.4	2,198	No	No
12	2.3	7.25	7	0.5	2,210	No	No

^*^6 months after surgery; ^**#**^3 months after surgery.

Eye pain peaked at day 1 and day 2 post-treatment. On day 1 after treatment, 11 patients (91.7%) had worse-than-baseline eye pain, among which 9 patients (75%) required oral pain medication. On day 2 after treatment, 5 patients (41.7%) had worse-than-baseline eye pain, among which 1 patient (8.3%) required oral pain medication. On day 4 after treatment, 11 patients (91.7%) had better-than-baseline eye pain and 1 patient (8.3%) had eye pain that remained at the same level as baseline, indicating that eye pain was relieved. Chemical conjunctivitis was not observed.

### 3.3 Clinical outcomes after DLKP

**Figure 3:**
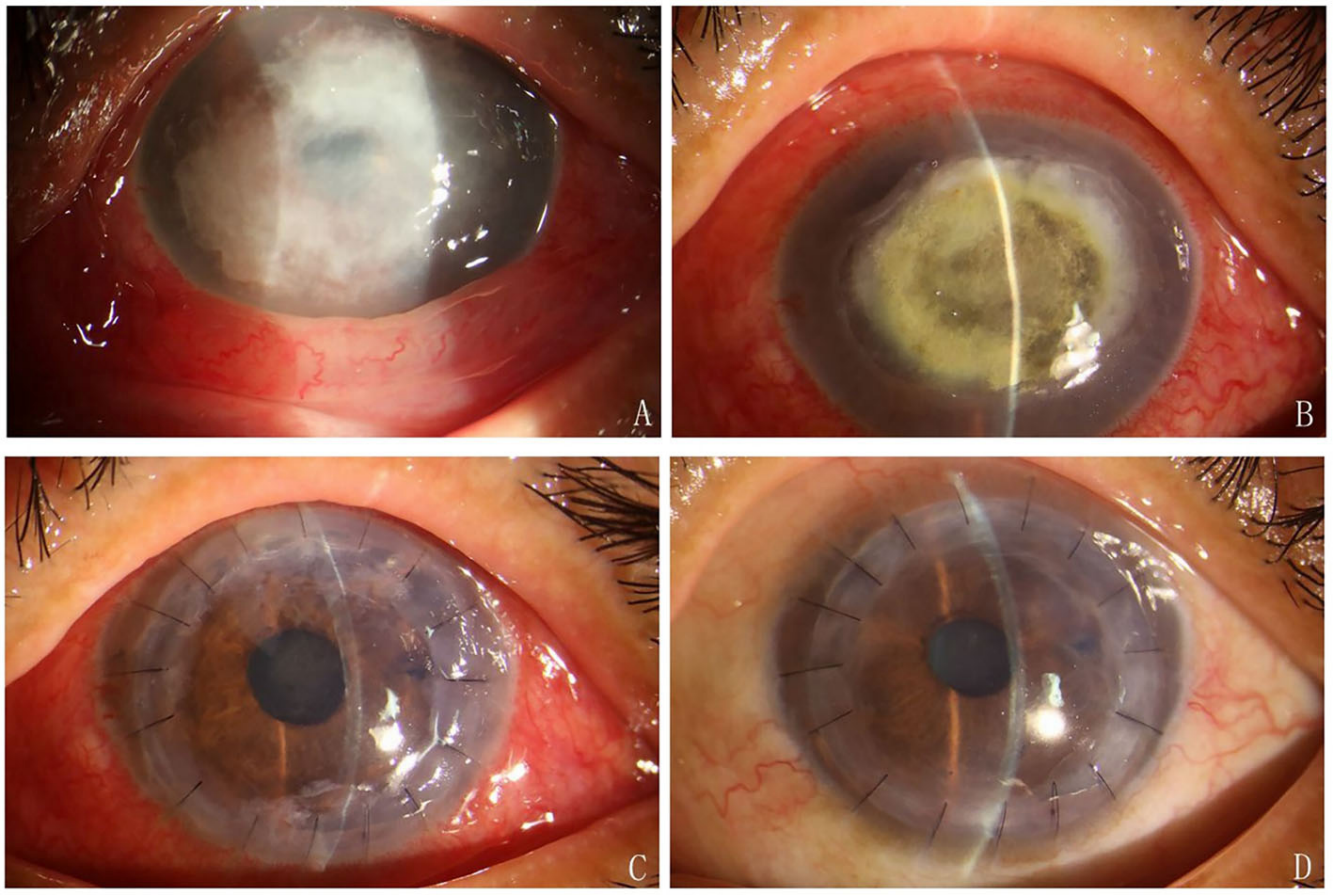
**(A)** A large corneal ulcer with feathery margins. **(B)** LASN was undergoing. **(C)** The graft was clear on day 7 after the DLKP. **(D)** 3 months after DLKP.

Outcomes after DLKP are shown in Table 2 and Figure 3. The median size of the donor graft was 7.60 ± 0.65 mm (ranging from 6.75 to 9.0 mm). During the follow-up period, no fungal recurrence was observed. No loose suture was observed in all patients within 3 months after corneal transplantation. At 6 months of follow-up, median BCVA (LogMAR) ranged from 0.9 to 0.3 with an average of 0.56 ± 0.19. The BCVA of all patients was improved after the operation. The mean corneal ECD ranged from 1,959 to 2,340 cells/mm^2^ with an average of 2,166.83 ± 119.75 cells/mm^2^.

## 4 Discussion

Recurrence of fungal keratitis after keratoplasty is a common complication. Currently, a variety of therapeutic approaches have been used to manage the recurrence of fungal keratitis after therapeutic keratoplasty (11). To avoid recurrence, the surgeon should try to completely remove the lesion as much as possible and cut off the area as long as infection is suspected during the surgery process. After surgery, antifungal medications (natamycin or voriconazole) are administered as eye drops or through intravenous subconjunctival injection. However, there are few reports on the treatment methods before keratoplasty. Our preliminary results showed that LASN is effective, safe, and well tolerated by patients.

In this study, we showed that, after LASN the corneal ulcer area was reduced, the infiltration and edema around the ulcer were reduced with clear boundary, and the empyema was absorbed. Clinical signs were significantly improved after 7–9 days of LASN. Studies have shown that silver nitrate exhibits potent antifungal activity against corneal isolates of Fusarium and Aspergillus *in vitro* and may be superior to natamycin (12). One previous study on the antifungal activity of silver ions from silver nitrate solution showed that the silver ion exerts its antifungal effect by inducing detachment of the plasma membrane and damage to cytoplasmic organelles and had remarkable efficacy against *A. flavus* OC1, which can cause major fungal infections to human (13). The severity of FK and the resultant corneal damage or resolution can be attributed to the bioburden of the fungal pathogen (14). After 7–9 days of LASN, the number of hyphae was significantly reduced, as determined by confocal microscopy. Therefore, silver nitrate can quickly and effectively control the disease and can improve the therapeutic efficacy of DLKP.

One previous study showed that fungal recurrence after deep anterior lamellar keratoplasty was 3.2% (15). Another study showed that fungal recurrence was 47.05% (16). The depth of the corneal infiltration was very important. OCT can directly visualize and measure the thickness of the infiltrations (17). Corneal infiltration was imaged as a hyperreflective area in the corneal stroma on high-resolution anterior segment OCT (18). In this study, we measured the depth by OCT to ensure patients met the inclusion criteria. The clearness of the infected lesion's edge was also important as it reflects a surgeon's ability to correctly judge and remove the infected corneal tissue thoroughly during the process of keratoplasty (19). Feathery margins and satellite foci are the most characteristic clinical features of fungal keratitis. Blurring boundary and the irregular shape of the lesion affect the judgment on the boundary during corneal transplantation. In order to completely remove the lesion, a larger corneal graft is required for the transplantation. A larger graft provides an opportunity to remove larger infected tissue, but larger grafts can increase the incidence of graft rejection compared to smaller grafts. Recurrent fungal infection after penetrating keratoplasty can occur at various sites among which recipient bed had a recurrent rate of 85.2% (11). The morphological characteristics of the lesions are an important factor affecting fungal recurrence after LKP treatment for fungal keratitis. In our study, the ulcer boundary was clear after LASN, and corneal transplantation performed at this time might have a low risk of infection recurrence because of possibly adequate eradication of infectious tissue and smaller grafts used for corneal transplant surgery. During the follow-up period, no fungal recurrence was observed in 12 patients, and no graft rejection occurred.

Pre-operative hypopyon, a common characteristic of fungal keratitis, is an independent risk factor for graft failure (20). However, hypopyon is not a contraindication of lamellar keratoplasty. Raj et al. reported that lamellar keratoplasty was performed in 295 patients with fungal keratitis, of which 67 eyes (22.7%) had hypopyons and 53% of the hypopyons were negative in fungal culturing (21). When hypopyon occurs, anterior chamber washout is required during the operation, and the risk of postoperative hyphema and exudation can be increased. In this study, the hypopyon disappeared before DLKP, intraoperative flushing of the anterior chamber was avoided, and the postoperative complications were reduced. At the same time, the risk of fungal recurrence after surgery was reduced.

Microbial infection of a corneal transplant is a severe complication, and the consequence can be devastating. Exposed, loose, or broken sutures are risk factors for infection (22). Suture-related problems (loose/broken sutures or exposed knots) occur in 60% of the patients in the group with infections, while they occur only in 38.9% of patients in the control group (*p* < 0.01) (23). Suture-related complications can be divided into intraoperative and postoperative. Intraoperative complications were heavily influenced by the surgeon's surgical skills; therefore, a novel surgeon or a surgeon in training should pay attention to the most common problems facing corneal transplant sutures (24). Loosening of sutures may develop after keratoplasty for four main reasons: (1) a tight suture becomes loose due to deturgescence of the graft; (2) insufficient re-epithelization at the suture tract resulting in potential microbial infection and stromal loss; (3) biodegradation of the suture material; (4) absence/loss of Bowman's in keratoconus resulting in cheese wiring of the suture (25).

The suture erosions can occur at any time after surgery ranging from weeks to months and sometimes years. A higher incidence of loose sutures occurs within 3 months after keratoplasty for infectious keratitis (26). However, suture loosening occurs earlier (usually 1 month) after keratoplasty for infectious keratitis, which could be due to implant bed edema in patients with infectious keratitis (27). Cases of penetrating keratoplasty performed for microbial keratitis often have severe ocular surface inflammation and are susceptible to suture-related problems, thus necessitating surgical intervention. This symptom is often observed during the early postoperative period (28). This study showed that, after treatment with silver nitrate, corneal edema around the ulcer was reduced and the risk of postoperative suture loosening was decreased. No loose sutures were observed in all 12 patients in the 3 months of follow-up period.

Ocular pain was a major complication, which occurred mainly 2 days after LASN and was successfully treated with oral painkillers. Pain medications were also required during the application of silver nitrate in the nasal area (29). Chemical conjunctivitis has been a common complication in previous studies. In this study, the application of cotton sticks could have enhanced silver nitrates at the targeted sites, thereby improving treatment efficacy and avoiding damage to the cornea and conjunctiva. Rinsing with sterile water rather than sodium chloride significantly reduced the incidence of chemical conjunctivitis (30). After the treatment, the patient received conjunctival sac irrigation using sterile water immediately. Fluorescein staining was performed for all patients, and no coloration was observed in the cornea surrounding the ulcer and conjunctiva. In this study, silver nitrate did not cause serious adverse effects on the cornea and conjunctiva.

Our pilot study showed that LASN plays an important role before DLKP. During the follow-up period, no fungal recurrence and no loose suture were observed in all the patients. Corneal grafts were well transparent, and all patients had improved postoperative vision. LASN as an adjuvant treatment before DLKP might be a promising therapeutic strategy.

There were several limitations of this study. First, this study has a small sample size and lack of control groups; Second, this study lacks a standard, clearly defined dose of LASN. Third, growth patterns of fungal pathogens may be an important factor for recurrence after DLKP. *Fusarium* was the main fungal pathogen detected in all patients. *Fusarium* has a horizontal growth pattern and it was relatively effective to be treated. For patients infected with *Aspergillus* (hyphae growing vertically), the therapeutic efficacy was difficult to determine and further studies were required.

## Data availability statement

The original contributions presented in the study are included in the article/supplementary material, further inquiries can be directed to the corresponding authors.

## Ethics statement

The study protocol was approved by the Institutional Review Board of Ethics Committee of Jinan Second People's Hospital (Jinan Eye Hospital) (No. JNEYE20210205). The studies were conducted in accordance with the local legislation and institutional requirements. Written informed consent for participation in this study was provided by the participants' legal guardians/next of kin.

## Author contributions

GD: Conceptualization, Investigation, Methodology, Writing—original draft. XG: Conceptualization, Investigation, Methodology, Writing—original draft. YT: Data curation, Resources, Visualization, Writing—original draft. ZH: Data curation, Resources, Visualization, Writing—original draft. XW: Data curation, Writing—original draft. CZ: Project administration, Supervision, Validation, Writing—review & editing. AD: Supervision, Writing—review & editing.
